# Investigating the Effects of Long-Term Ambient Air Storage on the Sliding Properties of N-Alloyed MoSe_2_ Coatings

**DOI:** 10.3390/nano15060414

**Published:** 2025-03-07

**Authors:** Talha Bin Yaqub, Irfan Nadeem, Muhammad Aneeq Haq, Muhammad Yasir, Albano Cavaleiro, Mitjan Kalin

**Affiliations:** 1 Laboratory for Tribology and Interface Nanotechnology (TINT), Faculty of Mechanical Engineering, University of Ljubljana, Askerceva 6, 1000 Ljubljana, Slovenia; 2 Department of Mechanical Engineering, CEMMPRE, ARISE, University of Coimbra, Rua Luis Reis Santos, 3030-788 Coimbra, Portugal; albano.cavaleiro@dem.uc.pt; 3 School of Chemical and Materials Engineering (SCME), National University of Sciences and Technology (NUST), H-12, Islamabad 46000, Pakistan; aneeq.haq@scme.nust.edu.pk; 4 Department of Fire Safety Engineering and Management ICEM, Muscat 2511, Oman

**Keywords:** MoSeN coatings, storage life cycle, solid lubricant, friction and wear, aerospace industry

## Abstract

Transition metal dichalcogenide coatings have emerged as potential candidates for terrestrial and aerospace mobility applications. Among these, the alloyed MoSe_2_ coatings have displayed promising results while sliding in diverse environments. N-alloyed Mose_2_ coatings provide the additional benefit of overcoming the impact of PVD compositional variations on dry sliding, making them promising solid lubricants for mobility-sector applications. However, the impact of long-term storage has never been investigated for this rarely studied solid-lubricant system. This study investigates the tribological performance of direct current magnetron sputtered MoSeN coatings after 40 months of storage in an ambient atmosphere. Sliding tests were conducted under conditions consistent with pre-storage conditions. The results showed that coatings with 0 at. %, 22 at. %, 33 at. %, and 35 at. % N-alloying exhibited COF values nearly identical to the pre-storage results, with only a negligible increase in ~0.01. Similarly, all coatings displayed specific wear rates in the range of 10^−7^, aligning with earlier findings. The obtained results show that the sliding performance of MoSeN coatings does not deteriorate over time, highlighting their suitability for critical aerospace applications, where components and assembled parts may be stored for years before launching into space or in actual applications.

## 1. Introduction

In past few decades, considerable efforts have been made to develop a solid lubricant-based coating system that can mitigate the adverse effects being faced due to liquid lubrication in both terrestrial and non-terrestrial atmospheres [1,2,3]. Thus, the modern research is aggressively focused on the optimization of tribo-mechanisms for reduction in friction and wear in mobility sectors via a sustainable solid lubricant coating system. This research has brought forth promising results, and a few of these coatings are already in use with continuing efforts for their efficiency enhancement [4,5,6].

Moreover, similar efforts resulted in emergence of a potentially suitable systems based on transition metal dichalcogenide (TMD) coatings [7,8]. Basically, TMDs have a layered structure which helps to provide easy shear properties and thus fulfill the requirements of low friction materials [9,10]. TMDs have been utilized since 1960s [11]. Significant efforts have been made to enhance the properties of TMD based coatings for efficient sliding. These include preparation by ion-beam-assisted depositions, RF, DC, Diode, and magnetron sputtering sources [12,13,14,15,16,17]. Similarly, refs. [18,19,20,21,22] report the efforts carried out to prepare TMD coatings with enhanced characteristics after alloying of metals and their oxides or preparing them in the form of multi-layers. Nevertheless, the reports published in ref. [8,14,23] provide thorough understanding of various approaches that were used to synthesize optimized TMD coatings until the late 19th century, as well as the critical performance results in various sliding conditions. However, despite promising results, developing a suitable solid lubricant that can provide long-term lubrication was an issue. The sputtered coatings based on pure TMDs are actually not capable of resisting wear due to their low load-bearing capabilities [24,25]. Additionally, the porous morphology is not capable of resisting moisture and similar environmental attacks, resulting in adverse sliding performance and increments of COF [22,26,27]. In an effort to combine different types of 2D materials for enhanced sliding efficiency and to overcome the adverse effects of pure TMDs, Voevodin combined the pure WS_2_ coatings with WC and DLCs to enhance the environmental resistance and load-bearing capabilities of these coatings [28]. Productive results were obtained, and the coating sliding performance improved substantially. This was followed by thorough investigations of these coatings where efforts were made that involved alloying with different metals [29,30,31,32,33] and non-metals [34,35,36,37] explored to enhance the compactness, reduce porosity, and improve load-bearing capabilities for effective enhancement in wear resistance. The efforts resulted in fruitful results, with the best performance being achieved with both C and N alloying of different TMDs (e.g., MoS_2_, WS_2_, and MoSe_2_).

Among these coating systems, the direct current (DC) magnetron sputtered N-alloyed MoSe_2_ (i.e., MoSeN) system has been recently developed and reported by current authors [38]. These MoSeN coatings displayed promising sliding properties in ambient air and proved suitable for 3D complex parts as they efficiently overcame the impacts of compositional variations on the sliding efficiency typically faced in PVD sputtered coatings.

Despite the development of different coating systems that can provide low friction in different environments, the scientific community pays less attention to a crucial aspect, i.e., the storage life analysis of these under-development coatings. In the urge to develop a long-lasting and durable solution, it is critically important to see the performance of solid lubricant coatings over time. This is vital as the coatings may perform above expectations soon after synthesis; however, long-term storage may degrade their efficient sliding performances. There are various means of degradation of the properties like (i) the storage conditions and atmosphere, (ii) the moisture present in the ambient atmosphere, and (iii) the temperature fluctuations that are experienced in seasonal variations, etc. All these factors can contribute to the changes in surface properties like impurities introduction, built-up of atomic layer oxides, or modifications of the structure of the coatings due to corrosion or environmental degradation (e.g., due to moisture attack), etc. It is very common in industries that coated parts often remain in inventory for years before being employed in machines. Thus, understanding consistent efficiency, regardless of the time elapsed before use, is crucial. As per the best knowledge of the present authors, only a few studies report the analysis of TMD coatings after some years of storage. For example, a study by Gustavsson et al. [39] mentioned only the analysis of chemical composition and structure of Mo-Se-C coatings after a gap of 5 years, and no significant variation was achieved in these properties. Similarly, the aging effects of TMDs and the growth of thin oxide layers have been reported in various studies referred to in refs. [40,41,42].

To the best of our knowledge, the impact of long-term storage on sliding properties of alloyed-TMD coatings, especially Mo-Se-N, has never been explored. Thus, considering the need to assess the sliding properties after long-term storage, MoSeN coatings deposited by current authors in April 2021 were re-evaluated after 40 months of storage and compared with previously measured results. Over a period of 40 months, these coatings were stored under ambient air conditions in plastic bags without any additional precautions. Therefore, the aim of this study is to emphasize the critical need to assess the storage durability of the coatings, particularly for demanding applications in industries such as automotive and aerospace, where consistent performance over an extended (storage) period is crucial.

## 2. Experimental Procedure

The coatings were synthesized in a semi-industrial scale deposition chamber by a N_2_ gas reactive magnetron sputtering of a DC-powered MoSe_2_ target in an Ar atmosphere. The depositions were carried out at a fixed chamber pressure of 0.27 Pa. The N_2_ gas flow was varied from 0 to 5 sccm to allow the deposition of a series of coatings with varying nitrogen content. Besides the MoSe_2_ target, a Cr target was also utilized for the deposition of the Cr interlayer and Cr-MoSeN gradient layer. The total deposition time for interlayer and gradient layers was 15 min each while the final coating deposition time was varied to achieve coatings of approx. 2 µm thickness. Mirror-polished DIN 100Cr6 (AISI-52100) steel (Ø 25 × 7 mm) samples having roughness (Ra) less than 0.02 µm and ~9.8 GPa hardness were used as substrates. The substrates were ultrasonically cleaned using ethanol for 15 min before deposition. A set of four coatings, alloyed with 0–35 at. % N, were deposited and stored for 40 months. The results of tests performed soon after coating deposition (i.e., before storage) are reported in ref. [38].

Post-storage sliding tests were carried out using a reciprocating sliding tribometer (UMT-2, Bruker, Billerica, MA, USA) for 1200 s under ambient conditions (RH = ~30–35%) against a 6 mm 100Cr6 ball. The stroke length and sliding frequency were fixed to 5 mm and 10 Hz, respectively. The tests were performed at an average sliding speed of 0.1 m/s during the stroke and an applied normal load of 3.5 N, corresponding to an initial Hertzian contact pressure of 1 GPa. These parameters were chosen to replicate the sliding conditions used in the previous study conducted 40 months earlier [38]. The tests were repeated three times, and the COF shown was systematically calculated based on the evolutions. This means that initially, the results from three repetitions were obtained. Then, the steady state regions of the COF evolution of each repetition were selected to calculate the average COF and standard deviation of that repetition. Finally, the average steady state COF and respective standard deviations of all repetitions were used to calculate the final average COF and deviations (errors). Thus, the bar charts represent these final average values, and the comparison has been made among the achieved final COF values shown in the bar chart.

After testing, ball and disk worn regions were examined using a digital optical microscope (Hirox-HRX-01, Kyoto, Japan). For disks, the wear profiles at three different zones of wear track from each repetition were measured using a white-light interferometer (Bruker Contour GT-K0, Billerica, MA, USA). These wear profiles were used to calculate the worn area. The worn area and stroke length were then used to calculate the total wear volume. Finally, the wear volume, applied load, and sliding distances were used to calculate specific wear rates in mm^3^/Nm units. The average values were then calculated and reported in this work. Likewise, specific wear rates of the steel counterparts were determined by measuring the worn scar diameters observed under the optical microscope.

The as-deposited coatings and disk wear tracks were also analyzed in Raman spectroscopy using a confocal Raman microscope (Horiba Xplora Plus, Kyoto, Japan) for the investigation of the sliding-induced structural changes and the possible mechanism behind the frictional results obtained in this work. The Raman analysis was performed using a 512 nm laser and the acquisition conditions of 1200 grating, 6 repetitions of 15 secs each, and 90% filter were chosen to avoid damage to the coatings and wear tracks.

## 3. Results and Discussion

### 3.1. Fundamental Properties of the Coatings

**Figure 1:**
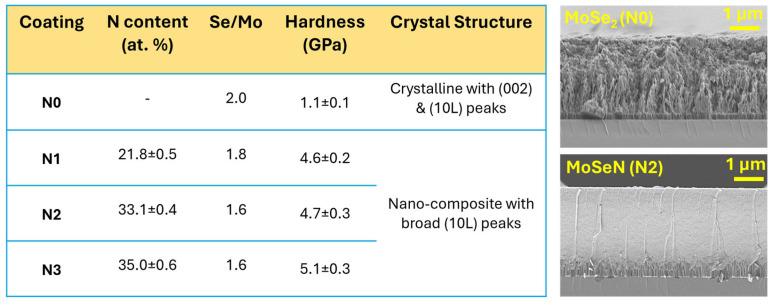
Overview of fundamental characteristics of MoSeN coatings—composition, crystal structure, and SEM morphology micrographs (N2 micrograph is shown as representative of all MoSeN coatings).

The fundamental characteristics of the coatings are detailed in ref. [38]. Briefly, the coatings contained N concentrations ranging from 0 to 35 at.%. This resulted in Se/Mo ratio ranging from 1.6 to 2.0, which decreased with increasing N content (Figure 1). The coatings are denominated as mentioned in Figure 1, i.e., N0, N1, N2, and N3 correspond to 0 at. % N, 21.8 at. % N, 33.1 at. % N, and 35 at. % N, respectively. The morphology of coatings transitioned from columnar and porous for pure coating to dense and compact with N-alloying. In terms of crystal structure, the pure coating (N0) exhibited crystalline peaks corresponding to the (002) basal planes as well as (100) and (10L) peaks. Whereas, N incorporation enhanced the amorphousness, and the coatings became nano-crystalline. The pure coating exhibited a hardness of 1.1 GPa, which increased to about 5 GPa with N addition (Figure 1). Overall, except for the pure coating, all other MoSeN coatings exhibited almost similar morphology, crystal structure, and hardness values, regardless of N content and Se/Mo ratios. These consistent properties, despite compositional variations, are advantageous for the industrial application of PVD sputtered coatings. This is because PVD sputtering is a highly line of sight deposition method [43], and, thus, the compositional variations for depositions on 3D parts is thus very common.

### 3.2. Tribological Results

**Figure 2:**
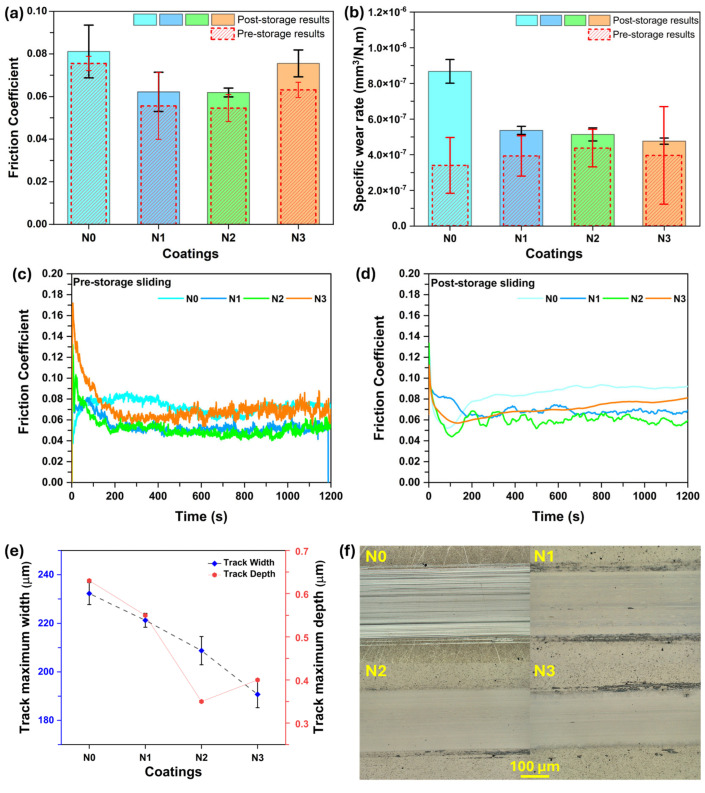
Tribological performance of MoSeN coatings, (**a**) post-storage average friction coefficients, (**b**) post-storage average specific wear rates, (**c**) pre-storage friction coefficients evolution, (**d**) post-storage friction coefficients evolution, (**e**) maximum wear-tracks width and depth from post-storage results, and (**f**) optical images of wear tracks from post-storage results.

The average COF results after 40 months of storage are shown in Figure 2a. For all coatings, initially, the COF decreased and then increased in the running-in phase to finally reach the steady-state sliding zone. The pure N0 coating exhibited an average steady-state COF value of 0.081. With N-alloying, the N1 coating showed a steady state average COF of 0.062. Similarly, the N2 coating’s average steady-state COF was 0.061. For N3 coating, the COF never reached a stable steady state zone, and it continued to vary. For example, in one of the repetitions, the minimum COF was around 0.055 (at 130 s), and then it continued to increase till the end of the test. Thus, for this coating, the average was calculated from the point of minimum COF till the end of the test. So, the average COF calculated was 0.076. The COF evolution comparison in pre- and post-storage tests is shown in Figure 2c and Figure 2d, respectively.

Thus, from the obtained results, it was observed that for N-alloyed coatings, the N3 coating displayed slightly unstable/fluctuating COF and slightly increased value. Overall, all coatings exhibited a minor increase in the COF in comparison to the results obtained immediately after deposition (40 months ago). It should be noted that this minor increase can be attributed to the build-up of a small oxidation layer on the very small vicinity of the coating surface during this long-storage duration. It is very common that the atmospheric oxygen may interact with the surface of materials during their storage in a normal atmosphere. However, this interaction has not shown a significant impact on the COF of the coatings even after 40 months, which seems impressive.

The specific wear rates of the coated disks are shown in Figure 2b. The N0 coating exhibited the highest specific wear rate of 8.67 × 10^−7^ mm^3^/Nm. The wear rate decreased to 5.36 × 10^−7^ mm^3^/Nm for N1 coating. With further increments in the N content, the specific wear rates further decreased to 5.13 × 10^−7^ mm^3^/Nm for N2 coating and 4.76 × 10^−7^ mm^3^/Nm for N3 coating. The higher specific wear rate of the N0 coatings compared to N-alloyed coatings is due to its porous morphology and the low load-bearing capability of pure sputtered TMD coatings. The porous morphology makes the pure TMD coatings more prone to degradation because of environmental attacks, e.g., moisture [44,45]. Basically, in such cases, the O or moisture from the atmosphere can passivate the vacant dangling bonds and result in increased friction and wear of the coatings. For the N-alloyed coatings, wear rates quite marginally decreased with increasing N. However, the decrease as compared to the pure coating is more. This decrease is attributed to compact morphology and more environmental attack resistance.

Similarly, the slightly higher specific wear rates of the coatings as compared to the pre-storage conditions may be attributed to the slight oxidation of the outer surface which can cause the formation of oxides that can create abrasiveness. Moreover, the more profound aspect is the fact that a different specific wear rate measurement mechanism, i.e., a 3D white light optical interferometer (in VXI mode), with a greater resolution was used in the current study, whereas a low-resolution stylus profilometry was utilized in previous work. In pre-storage measurements, stylus profilometry was employed, which has less sensitivity as compared to the white light interferometer used in the current study. Nonetheless, the wear rate still lies in the 10^−7^ range, which is a promising result.

Figure 2e displays that the maximum wear track depths observed for N0, N1, N2, and N3 coatings were 0.63, 0.58, 0.34, and 0.42 µm, respectively. The optical images of coating wear tracks are shown in Figure 2f. All tracks showed abrasive wear marks and displayed features distinct from the as-deposited coatings. As per the previous experience and reported results, the tracks were covered with adhered materials, most likely MoSe_2_ tribolayers. The degree of coverage varied across coatings, with coverage decreasing with increasing nitrogen content. This decrease is directly related to the Se/Mo ratio, with higher N content reducing the availability of MoSe_2_ for tribolayer formation and coverage.

**Figure 3:**
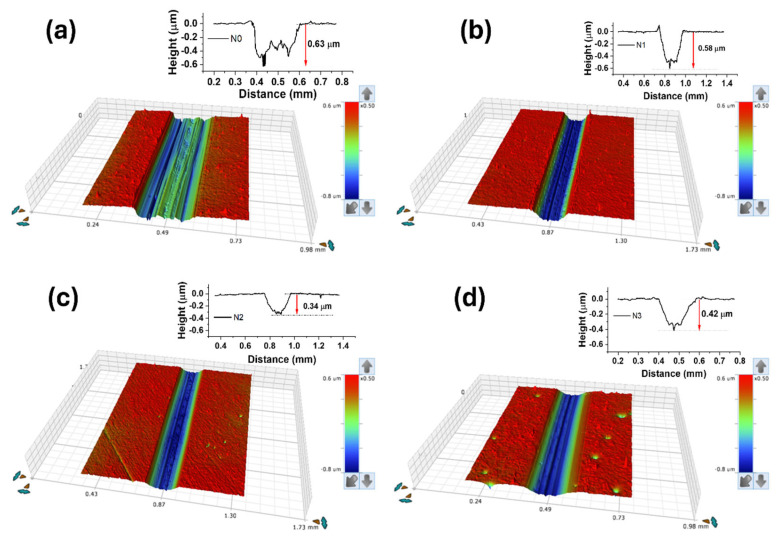
Three-dimensional surface topography and 2D profiles of wear tracks, (**a**) N0 coating, (**b**) N1 coating, (**c**) N2 coating and, (**d**) N3 coating.

Similarly, the 3D surface topography of wear tracks along-with 2D profiles are presented in Figure 3. Agreeing with the specific wear rate results, the pure reference coating displayed the highest wear and this decreased with the introduction of N in the coatings. It is clear that the sliding was always taking place in the outer coating, and it never reached the interlayer, even for the highest wear track depth zone. Moreover, the 3D topography and 2D profilometer results are in accordance with the optical images displayed in Figure 2f.

**Figure 4:**
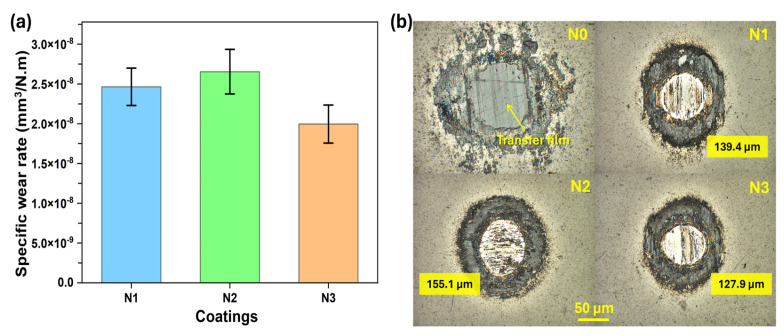
Sliding results—(**a**) specific wear rates of counter bodies (balls) and (**b**) optical images of ball wear scars.

The specific wear rates and wear scars of the steel counterparts are shown in Figure 4a,b. The N0 coating steel counterpart showed no wear and was covered by a thick layer of adhered material, which correlates with the high disk wear rate. Basically, the N0 coating was softer and, due to the high wear of the soft coating, enough material was transferred to protect the counterpart. Specific wear rate for the N1 coating counterpart was 2.48 × 10^−8^ mm^3^/Nm, which minorly increased to 2.72 × 10^−8^ mm^3^/Nm for the N2 coating counterpart while it then decreased to 2.14 × 10^−8^ mm^3^/Nm for the N3 coating counterpart.

### 3.3. Raman Spectroscopy Analysis of Wear Tracks

**Figure 5:**
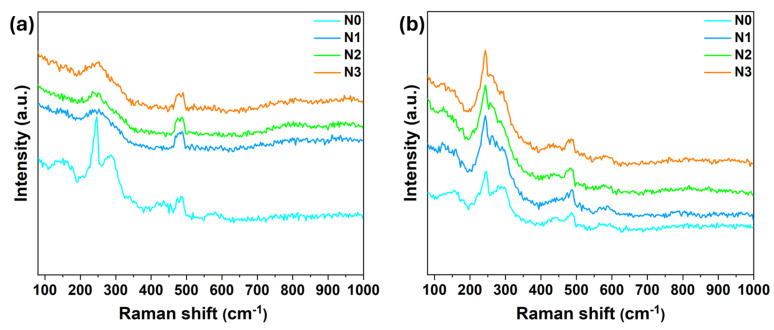
Raman spectra acquired on the coated disks. (**a**) As-deposited coating zone, and (**b**) inside the wear tracks (MoSe_2_ peaks region = 150–350 cm^−1^).

The Raman spectroscopy analysis of the wear tracks was performed to investigate the reasons behind the low friction of the coatings as well as the role of TMD phase during sliding. The analysis was performed on the as-deposited surfaces and in the middle of the wear tracks. The results for the as-deposited coatings and wear tracks are shown in Figure 5a and Figure 5b, respectively. From the as-deposited coatings zone results, it is clear that the pure N0 coating displayed well defined crystalline peaks of MoSe_2_ while the N-alloyed as-deposited coatings did not exhibit well-defined peaks. These observations are consistent with the crystal structure results (see Figure 1). However, the analysis performed inside the wear tracks revealed distinct crystalline MoSe_2_ peaks in all coatings. This increase in crystallinity after sliding indicates tribo-induced crystallization as well as chemical changes in the contact zone. This crystallization results in the formation of an easy shear tribolayer of MoSe_2_, driven by reorientation and transfer layer mechanisms, as observed in MoSeC coatings [46]. Thus, in agreement with refs. [46,47], the formation of MoSe_2_ tribolayers is the key to low friction in the present case. These Raman spectroscopy results thus confirm the hypothesis mentioned above in the optical image results that the tracks features are different than the as-deposited coatings and the tracks are covered with an adhered material (transfer film).

In summary, it is clear that the 40 months ambient air storage had no significant impact on the performance or shelf-life of the coatings. All coatings except N0 exhibited similar friction and wear behavior with very minor increase in comparison to the results obtained immediately after deposition (40 months ago). These coatings remained in an ambient atmosphere with seasonal variations in humidity and minor temperature fluctuations in winters and summers. Thus, the results depict a minor difference in ~0.01 in COF and within 10^−7^ mm^3^/Nm range wear rates. Moreover, the slight differences in specific wear rates can be attributed to the use of a white-light interferometer in this study, offering higher resolution than the stylus profilometer used in the pre-storage tests. Therefore, these coatings are quite stable and suitable for industrial applications requiring long-term storage. Furthermore, it is noteworthy that the consistent sliding performance of N-alloyed coatings, regardless of compositional variations, further reinforces the previous findings. The ability to maintain a similar COF across different compositions is a significant achievement, addressing the drawbacks of PVD sputtering and making MoSeN coatings particularly favorable for industries relying on PVD sputtering technology.

## 4. Conclusions

This research investigates the sliding characteristics of MoSeN coatings after a long-term storage of 40 months. The MoSeN coatings with N content ranging from 0 to 35 at. % were deposited using reactive DC magnetron sputtering. The sliding tests performed after 40 months of storage showed results very similar to pre-storage tests and only minor variations were observed. The pure N0 coating displayed a COF of 0.081 and a wear rate of 8.67 × 10^−7^ mm^3^/Nm, while all MoSeN coatings displayed COF values between 0.061 and 0.076, with wear rates ranging from 4.76 to 5.35 × 10^−7^ mm^3^/Nm. Two-dimensional profiles and 3D topography results demonstrated that the coating did not delaminate, and the sliding occurred within the outer coating. Similarly, Raman spectroscopy analysis of virgin coating and wear tracks concluded that crystallized MoSe_2_ signals were detected only inside the wear tracks and not from virgin MoSeN coating surfaces. This proved that the low friction characteristics are governed by MoSe_2_ tribolayers. These findings demonstrate that globally, the coatings’ performance is unaffected by long-term storage, making them suitable for industries requiring extended storage periods before implementation.

## Data Availability

Data is contained within the article.

## References

[1] Fitch E.C. (1992). Fluid Chemical Stability. Proactive Maintenance for Mechanical Systems.

[2] Ciulli E., Quaglia G., Gasparetto A., Petuya V., Carbone G. (2022). Tribology and Sustainable Development Goals.

[3] Technical Committee of Petroleum Additive Manufacturers in Europe (2007). Lubricant Additives and the Environment.

[4] Donnet C., Erdemir A. (2008). Diamond-like Carbon Films: A Historical Overview.

[5] Hilton M.R., Fleischauer P.D. (1992). Applications of Solid Lubricant Films in Spacecraft. Surf. Coat. Technol..

[6] Aouadi S.M., Gao H., Martini A., Scharf T.W., Muratore C. (2014). Lubricious Oxide Coatings for Extreme Temperature Applications: A Review. Surf. Coat. Technol..

[7] Lansdown A.R. (1999). Molybdenum Disulphide Lubrication.

[8] Roberts E.W. (1990). Thin Solid Lubricant Films in Space. Tribol. Int..

[9] Yaqub T.B., Vuchkov T., Evaristo M., Cavaleiro A. (2019). DCMS Mo-Se-C Solid Lubricant Coatings—Synthesis, Structural, Mechanical and Tribological Property Investigation. Surf. Coat. Technol..

[10] Yaqub T.B., Al-rjoub A., Cavaleiro A., Fernandes F. (2022). Exploring the Industrial Implementation of W-S-N Coatings: A Detailed Study of the Synthesis, Compositional, Structural, Mechanical and Multi-Environment Lubrication Properties. J. Mater. Res. Technol..

[11] Voevodin A.A., Muratore C., Aouadi S.M. (2014). Hard Coatings with High Temperature Adaptive Lubrication and Contact Thermal Management: Review. Surf. Coat. Technol..

[12] Bolster R.N., Singer I.L., Wegand J.C., Fayeulle S., Gossett C.R. (1991). Preparation by Ion-Beam-Assisted Deposition, Analysis and Tribological Behavior of MoS2 Films. Surf. Coat. Technol..

[13] Sliney H.E. (1982). Solid Lubricant Materials for High Temperatures—A Review. Tribol. Int..

[14] Roberts E.W. (1989). Ultralow Friction Films of MoS2 for Space Applications. Thin Solid Films.

[15] Stewart T.B., Fleischauer P.D. (1982). Chemistry of Sputtered Molybdenum Disulfide Films. Inorg. Chem..

[16] Sekine T., Izumi M., Nakashizu T., Uchinokura K., Matsuura E. (1980). Raman Scattering and Infrared Reflectance in 2H-MoSe2. J. Phys. Soc. Jpn..

[17] Bichsel R., Levy F. (1984). Morphological and Compositional Properties of MoSe2 Films Prepared by R.F Magnetron Sputtering. Thin Solid Films.

[18] Mikhailov S., Savan A., Pflüger E., Knoblauch L., Hauert R., Simmonds M., Swygenhoven H. (1998). Van Morphology and Tribological Properties of Metal (Oxide)–MoS2 Nanostructured Multilayer Coatings. Surf. Coat. Technol..

[19] Wahl K.J., Dunn D.N., Singer I.L. (1999). Wear Behavior of Pb–Mo–S Solid Lubricating Coatings. Wear.

[20] Simmonds M.C., Savan A., Van Swygenhoven H., Pflüger E., Mikhailov S. (1998). Structural, Morphological, Chemical and Tribological Investigations of Sputter Deposited MoSx/Metal Multilayer Coatings. Surf. Coat. Technol..

[21] Spalvins T. (1984). Frictional and Morphological Properties of Au-MoS2 Films Sputtered from a Compact Target. Thin Solid. Films.

[22] Hilton M.R. (1994). Fracture in MoS2 Solid Lubricant Films. Surf. Coat. Technol..

[23] Fusaro R.L. (1978). Lubrication and Failure Mechanisms of Molybdenum Disulfide Films.

[24] Polcar T., Cavaleiro A. (2011). Self-Adaptive Low Friction Coatings Based on Transition Metal Dichalcogenides. Thin Solid Films.

[25] Fu Y., Wang Q., Guo L., Zhao X., Jiang D., Gao X., Weng L., Sun J., Hu M., Wang D. (2023). Interlayer Texturing for Improving the Tribological Property in Vacuum of Highly Crystallized Molybdenum Disulfide Film. Vacuum.

[26] Wang D.-Y., Chang C.-L., Ho W.-Y. (1999). Microstructure Analysis of MoS2 Deposited on Diamond-like Carbon Films for Wear Improvement. Surf. Coat. Technol..

[27] Voevodin A.A., Zabinski J.S. (2005). Nanocomposite and Nanostructured Tribological Materials for Space Applications. Compos. Sci. Technol..

[28] Voevodin A.A., O’neill J.P., Zabinski J.S. (1999). Nanocomposite Tribological Coatings for Aerospace Applications. Surf. Coat. Technol..

[29] Duan Z., Zhao X., Nai Z., Qiao L., Xu J., Wang P., Liu W. (2019). Mo-S-Ti-C Nanocomposite Films for Solid-State Lubrication. ACS Appl. Nano Mater..

[30] Bülbül F., Efeoğlu İ. (2016). Synergistic Effect of Bias and Target Currents for Magnetron Sputtered MoS2-Ti Composite Films. Mater. Test..

[31] Chien H., Ma K., Vattikuti S.V.P., Kuo C., Huo C., Chao C. (2010). Tribological Behaviour of MoS2/Au Coatings. Thin Solid Films.

[32] Scharf T.W., Goeke R.S., Kotula P.G., Prasad S.V. (2013). Synthesis of Au-MoS2 Nanocomposites: Thermal and Friction-Induced Changes to the Structure. ACS Appl. Mater. Interfaces.

[33] Zekonyte J., Cavaleiro A., Polcar T. (2014). Frictional Properties of Self-Adaptive Chromium Doped Tungsten-Sulfur- Carbon Coatings at Nanoscale. Appl. Surf. Sci..

[34] Zekonyte J., Polcar T. (2015). Friction Force Microscopy Analysis of Self-Adaptive W—S—C Coatings: Nanoscale Friction and Wear. ACS Appl. Mater. Interfaces.

[35] Cao H., De Hosson J.T.M., Pei Y. (2017). Effect of Carbon Concentration and Argon Flow Rate on the Microstructure and Triboperformance of Magnetron Sputtered WS2/a-C Coatings. Surf. Coat. Technol..

[36] Isaeva L., Sundberg J., Mukherjee S., Pelliccione C.J., Lindblad A., Segre C.U., Jansson U., Sarma D.D., Eriksson O., Kádas K. (2015). Amorphous W-S-N Thin Films: The Atomic Structure behind Ultra-Low Friction. Acta Mater..

[37] Seynstahl A., Köbrich M., Rosnitschek T., Göken M., Tremmel S. (2024). Enhancing the Lifetime and Vacuum Tribological Performance of PVD-MoS2 Coatings by Nitrogen Modification. Surf. Coat. Technol..

[38] Yaqub T.B., Fernandes F., Al-Rjoub A., Cavaleiro A. (2022). Mo-Se-N Dry Lubricant Coatings as a Universal Solution for Protecting Surfaces of Complex 3D Parts. Mater. Lett..

[39] Gustavsson F., Jacobson S., Cavaleiro A., Polcar T. (2013). Frictional Behavior of Self-Adaptive Nanostructural Mo-Se-C Coatings in Different Sliding Conditions. Wear.

[40] Gao J., Li B., Tan J., Chow P., Lu T.M., Koratkar N. (2016). Aging of Transition Metal Dichalcogenide Monolayers. ACS Nano.

[41] Liu H., Han N., Zhao J. (2015). Atomistic Insight into the Oxidation of Monolayer Transition Metal Dichalcogenides: From Structures to Electronic Properties. RSC Adv..

[42] Rajput N.S., Kotbi A., Kaja K., Jouiad M. (2022). Long-Term Aging of CVD Grown 2D-MoS2 Nanosheets in Ambient Environment. Npj Mater. Degrad..

[43] Vuchkov T., Evaristo M., Yaqub T.B., Cavaleiro A. (2020). The Effect of Substrate Location on the Composition, Microstructure and Mechano-Tribological Properties of W-S-C Coatings Deposited by Magnetron Sputtering. Surf. Coat. Technol..

[44] Voevodin A.A., O’Neill J.P., Prasad S.V., Zabinski J.S. (1999). Nanocrystalline WC and WC/a-C Composite Coatings Produced from Intersected Plasma Fluxes at Low Deposition Temperatures. J. Vac. Sci. Technol. A Vac. Surf. Film..

[45] Polcar T., Cavaleiro A. (2011). Review on Self-Lubricant Transition Metal Dichalcogenide Nanocomposite Coatings Alloyed with Carbon. Surf. Coat. Technol..

[46] Yaqub T.B., Vuchkov T., Bruyère S., Pierson J.F., Cavaleiro A. (2022). A Revised Interpretation of the Mechanisms Governing Low Friction Tribolayer Formation in Alloyed-TMD Self-Lubricating Coatings. Appl. Surf. Sci..

[47] Yaqub T.B., Bruyere S., Pierson J.F., Vuchkov T., Cavaleiro A. (2020). Insights into the Wear Track Evolution with Sliding Cycles of Carbon-Alloyed Transition Metal Dichalcogenide Coatings. Surf. Coat. Technol..

